# Green synthesis of MgO nanoparticles and its antibacterial properties

**DOI:** 10.3389/fchem.2023.1143614

**Published:** 2023-03-23

**Authors:** Rajeshwari B. Rotti, D. V. Sunitha, Ramya Manjunath, Arpita Roy, Shilpa Borehalli Mayegowda, A. P. Gnanaprakash, Saad Alghamdi, Mazen Almehmadi, Osama Abdulaziz, Mamdouh Allahyani, Abdulelah Aljuaid, Ahad Amer Alsaiari, Sami S. Ashgar, Ahmad O. Babalghith, Amal Ezzat Abd El-Lateef, Elshiekh B. Khidir

**Affiliations:** ^1^ Department of Physics, School of Applied Sciences, REVA University, Bangalore, India; ^2^ Department of Biotechnology, School of Applied Sciences, REVA University, Bangalore, India; ^3^ Department of Biotechnology, Sharda School of Engineering and Technology, Sharda University, Greater Noida, India; ^4^ CHRIST-Deemed to be University, Kengeri Campus, Bangalore, Karnataka, India; ^5^ Department of Studies in Physics, University of Mysore, Mysuru, India; ^6^ Laboratory Medicine Department, Faculty of Applied Medical Sciences, Umm Al-Qura University, Makkah, Saudi Arabia; ^7^ Department of Clinical Laboratory Sciences, College of Applied Medical Sciences, Taif University, Taif, Saudi Arabia; ^8^ Department of Microbiology, Faculty of Medicine, Umm Al-Qura University, Makkah, Saudi Arabia; ^9^ Medical Genetics Department, College of Medicine, Umm Al-Qura University, Makkah, Saudi Arabia

**Keywords:** anti-bacterial activity, defects, dosimetry, green synthesis, thermoluminescence

## Abstract

Magnesium oxide nanostructured particles (NP) were prepared using a simple solution combustion technique using different leaf extracts such as *Mangifera indica* (Mango - Ma), *Azadirachta indica* (Neem—Ne), and *Carica papaya* (Papaya—Pa) as surfactants. The highly crystalline phase of MgO nanostructures was confirmed by PXRD and FTIR studies for 2 h 500°C calcined samples. To analyze the characteristics of obtained material–MaNP, NeNP, and PaNP for dosimetry applications, thermoluminescence (TL) studies were carried out for Co-60 gamma rays irradiated samples in the dose range 10–50 KGy; PaNP and NeNP exhibited well-defined glow curve when compared with MaNP samples. In addition, it was observed that the TL intensity decreases, with increase in gamma dose and the glow peak temperature is shifted towards the higher temperature with the increase in heating rate. The glow peak was segregated using glow curve deconvolution and thermal cleaning method. Kinetic parameters estimated using Chen’s method, trap depth (E), and frequency factor (s) were found to be 0.699, 7.408, 0.4929, and 38.71, 11.008, and 10.71 for PaNP, NeNP, and MaNP respectively. The well-resolved glow curve, good linear behavior in the dose range of 10–50, KGy, and less fading were observed in PaNP as compared with MaNP and NeNP. Further, the antibacterial activity was checked against human pathogens such as *Escherichia coli, Staphylococcus aureus,* and *Pseudomonas aeruginosa.* A visible zone of clearance was observed at 200 and 100 μg/mL by the PaNP and NeNP, indicating the death of colonies by the nanoparticles. Therefore, PaNP nanomaterial is a potential phosphor material for dosimetry and antibacterial application compared to NeNP and MaNP.

## 1 Introduction

Nanoparticles are increasingly gaining attention in various domains of their applications in materials science, energy science, medicine, and biotechnology (Khan et al., 2019). These nanosized materials can be used in ceramics, catalysis, electronics, coatings, petrochemical products, metallic ceramics, and fiber boards. Nanoparticles are efficient in biological and medical applications viz., sensors, medical and optical devices, drug delivery, bacteriostatic, dye degradation, and, DNA labeling, etc., (Archana et al., 2021; Ananda et al., 2022). These applications are attributed to its large surface: volume ratio and smaller size. Synthesizing the nanostructured materials can be accomplished through physical, chemical, and bio-based methods (Krishna Moorthy et al., 2015; Rao and Singh, 2017; Ijaz et al., 2020). The different methods used for chemical synthesis such as precipitation, pyrolysis, micelle, hydrothermal and sol-gel processes render it toxic and a threat to the ecology and environment (Patil et al., 2021; Manjula et al., 2022). To minimize this negative effect on the environment, green synthesis becomes a savior. The biological raw materials for green synthesis can be microorganisms, algae, and plants. (Thunugunta et al., 2015; Praveen Kumar et al., 2020).

Green synthesis is one of the widely used, cost-efficient, non-toxic, and non-hazardous techniques for the preparation of simple metal oxides (Patil et al., 2021; Khandaker et al., 2022; Manjula et al., 2022; ShilpaMayegowda et al., 2022). Nanoparticles obtained by the green synthesis technique have diverse applications such as-anti-inflammatory, antimicrobial activity, effective drug delivery, bioactivity, tumor targeting, anti-cancer, and bio-absorption apart from biological applications they are also used in transistors, magnetic devices, photocatalysts, microelectronic devices, anticorrosive coatings, electrocatalysts and in powder metallurgy (Ijaz et al., 2020; Salem and Amr, 2021; Mayegowda et al., 2022a; Mayegowda et al., 2022b; Samuel et al., 2022; Wu and Mu, 2022). Different parts (root, stem, leaves, floor, seeds) of plant extracts are used for material preparation in the green synthesis technique. The extracts contain various phytochemicals, namely, flavonoids, alkaloids, phenolics, and other phytochemicals (Ugo et al., 2019), that are rich in carbon, hydrogen, and nitrogen compounds. The plant extract reacts with metal salts leading to the formation of nanoparticles of different sizes, shapes, and surface areas. (Shah et al., 2015).

Though the nanoparticles and metal oxide nanoparticles can be synthesized by seeds, bark, flowers, tuber, and root extracts, leaves are mostly preferred as a major resource for metabolites as they regenerate and they are also safe to use when compared to bacteria, algae, or any other plant tissue as plant leaves are preferably non-pathogenic (Ahmad et al., 2019). Also, a unique feature that adds on to the property is the leaf extracts have a faster reduction rate than algae or bacteria (Singh et al., 2018).

The plant extracts contain phytochemicals that play a vital role in the reduction of metal ions and form metal nanoparticles. The functional amino groups lead to a reduction of the metal oxide (Ugo et al., 2019). The oxygen present in the atmosphere or degrading phytochemicals reduces metal ions. Due to electrostatic attraction that mediates metal oxide formation and stabilizes the phytochemicals leading to agglomeration of particles. The superoxide is the main source of reactive oxygen species and is subdued by phenolic compounds with carboxyl and hydroxyl groups of the plant (Ugo et al., 2019). It is observed that high and low-weight phytochemicals, proteins, and starch mixtures present in the plant extract form metal nanoparticles due to the proteins present in the plant extract (Mittal et al., 2013; Ahmad et al., 2019; Ugo et al., 2019).

The term thermoluminescence (TL) is the combination of heat and emission of light. TL phenomenon is usually observed in crystalline material; the crystalline samples are pre-exposed to radiations and energy gets accumulated in the crystal samples, when crystals are heated, they re-emit energy in the form of light, absorbed previously by the crystalline material (Sunta, 2015).

Defects are the main cause of the generation of luminescence. Defects can be generated in two ways, viz., i) Structural defects created during material preparation and ii) defects created by adding doping material into the host material; in a few cases defects can also be created by exposure to radiations. The defects should be capable of holding the electrons and holes when exposed to ionizing radiations (Souadi et al., 2022). The trapped electrons/holes stored inside the material reach the optimized temperature by producing TL emissions in the crystalline material. TL material is more efficient when they have a high concentration of trap due to the creation of structural defects and by addition of impurities (Furetta, 2003).

TL dosimeters look forward to material that exhibits good linear dose-response, reusability, high sensitivity, and tissue equivalent materials (Bos, 2007). From the literature, the materials such as oxides, borates, fluoride, silicate, phosphate, and sulfate have shown very good TL behavior (Azorin, 2014; Patle et al., 2021; Sonsuz et al., 2022).

The diagnosis and treatment of numerous infectious diseases have been transformed in recent decades by nanomaterials and nanoparticles. Due to their numerous advantages, such as their high surface area to mass ratio, extremely small diameters, and special physical and chemical properties, antimicrobial nanoparticles are very promising. These can be the future alternative in the medical domain for bacterial infections (Rodrigues et al., 2019; Nafari et al., 2020; Raura et al., 2020). The nanoparticles synthesized using different metal oxides have proven to be toxic against bacterial cells as they can rapidly penetrate the cell wall and speeds up the formation of the ROS (reactive oxygen species) or degrades the enzymes in the bacterial cell wall and eventually lead to death (Mayegowda et al., 2022a; Mayegowda et al., 2022b). Many metal oxide nanoparticles synthesized *via* the green route, namely, ZnO-Nps are reported to be toxic against *Pseudomonas aeruginosa* and *Streptococcus mutans*. Au-Nps and Ag-Nps are reported to be toxic against *Bacillus subtilis*, *Bacillus cereus,* and *P. aeruginosa*. The bimetallic Ag/Cu and Cu/Zn bimetallic nanoparticles were studied for antibacterial activity against *Alcaligenes faecalis, Staphylococcus aureus, Citrobacter freundii, Klebsiella pneumoniae,* and *Clostridium perfringens.* (Merugu et al., 2021).

In a comparative study conducted by Sabeena and coworkers in 2022 (Sabeena et al., 2022), the antibacterial efficacy of CuO-Nps was tested against *S. aureus*, *Escherichia coli*, *Enterobacter*, *B. subtilis* and *P. aeruginosa* by the nanoparticles synthesized using the green route and chemical method. They concluded that green CuO-Nps showed higher antibacterial efficacy over chemically synthesized CuO-NPs.

Though there are many metal oxides such as ZnO, CuO, MgO, TiO, CdO, etc., amongst magnesium oxide has unique properties such optical, electronic, thermal, mechanical, and chemical properties compared to other metal oxides (Diachenko et al., 2016). It is a very stable and safe material to be used in different fields of applications and has high melting point, wide energy bandgap, high reactivity, and low heat capacity (Abdoul et al., 2020; Fatiqin et al., 2021). In the present paper, we have made an effort to do a comparative study of MgO Nanostructures prepared using mango, neem, and papaya leaf extracts on structural, morphological, luminescence, and antibacterial studies.

## 2 Materials and methods

Magnesium sulfate, used for nanoparticle synthesis, was procured from Fischer scientific (purity 99.5%). The healthy plant leaves–*Carica papaya* (Papaya-Pa), *Azadirachta indica* (Neem-Ne), and *Mangifera indica* (Mango-Ma) were collected from Yelahanka, Latitude N 13°6′ 11.7756 and Longitude E 77°36′15.044 of Bangalore, Karnataka.

**FIGURE 1:**
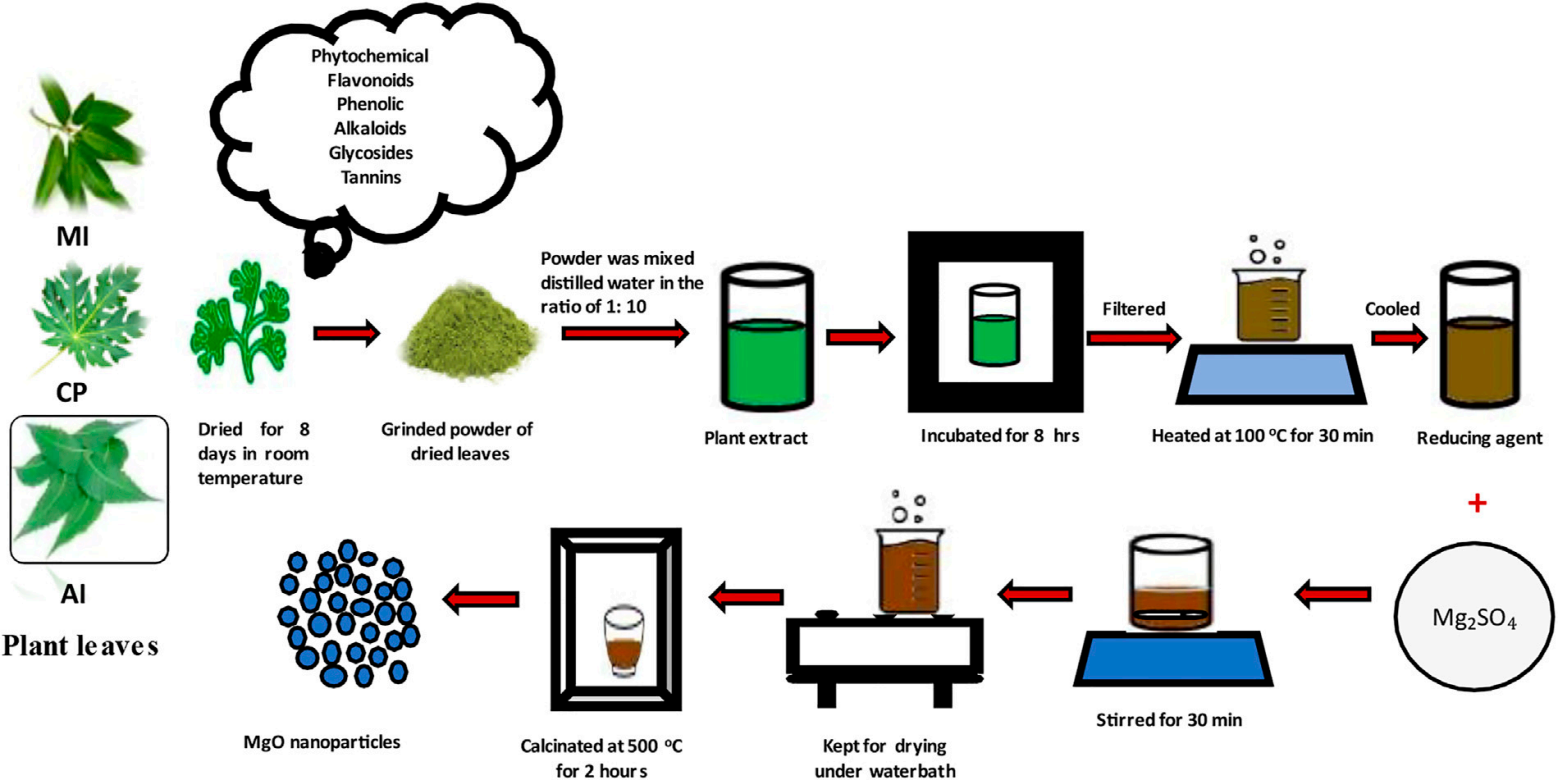
Procedure of preparation of magnesium oxide nanoparticles by simple solution method.

The healthy plant leaves were brought to the laboratory and washed with 1% sodium hypochlorite followed by distilled water to remove the dirt, wiped, and allowed to dry under shade for 8 days and powdered. 10 g of the leaf powder was mixed with 100 mL distilled water and placed in the shaker incubator at 130 rpm for 8 h. Post extraction, the solution was filtered and the filtrate was used for nanoparticle synthesis. 3 mM MgSO_4_ was added to 50 mL filtrate and boiled to 100°C for 30 m. Color change from green to brown was observed indicating the synthesis of nanoparticles. The brown color solid obtained by evaporation was calcined at 500°C and used for structural and morphological characterization (Ambika et al., 2015). The process of preparation MgO NP sample is illustrated in Figure 1.

### 2.1 Characterization and TL studies

The calcinated samples were subjected to structural, morphological, and luminescence studies by using different characterizing tools such as Powder X-ray diffraction (PXRD) patterns of MgO NPs were recorded using XPERT-3, PXRD instrument (Panalytic-make) using CuKα radiation of wavelength 1.54 nm, in the 2θ range of 20°–80°. Fourier transform infrared spectroscopy (FTIR) spectra were recorded in the range of 500–4,000 cm^−1^ using an alpha II spectrometer (Bruker -make). Surface morphology was analyzed using a Scanning electron (SEM) microscope (VEGA3 LMU). Absorption spectra were analyzed by Evolution 201 UV-visible spectrometer instrument (Thermo scientific–make) recorded in the range of 200–800 nm. Gamma irradiation in the dose range 10–50 KGy was done using Co-60 (BRIT-make) before TL studies and TL reading was recorded in the temperature range of 40°C–450°C at a heating rate of 2.5 Cs^−1^ using TL 1009I reader (Nucleonix-make).

### 2.2 Antibacterial activity

Preparation and dilution of nanoparticles for antibacterial activity: In this study, we used the gel diffusion method for checking antibacterial activity on three green synthesized NPs including papaya, neem, and mango as PaNP, NeNP, and MaNP, respectively. These NPs were processed together to avoid experimental unfairness. (Ambika et al., 2015).

Bacterial strains used: Three human pathogenic bacteria, Gram-negative *Pseudomonas aeruginosa* (gastrointestinal infections, several systemic infections) and *Escherichia coli* (diarrhea, pneumonia), and Gram-positive *Staphylococcus aureus* (abscesses, furuncles, and cellulitis) were procured from biotechnology training institute (Azymes Biosciences Pvt. Ltd., Bengaluru). In the current study, we have used three pathogens which are known to be nosocomial organisms found normally on the skin and mucous membranes and cause primary and secondary infections when contracted by a patient while under medical care. The green NPs are known to pose several bactericidal and bacteriostatic bioactive compounds like tannins, flavonoids, alkaloids, glycosides, and saponins (Jadimurthy et al., 2022).

The required concentration of NPs was diluted using autoclaved distilled water to obtain various concentrations (200, 100, 50, and 25 mg/mL).

The microbiological technique used:

Freshly procured bacterial strains were cultured by inoculating in nutrient broth (Hi-Media, Mumbai, India) and cultures were incubated for 18–24 h at 37°C these cultures were grown on nutrient agar (NA) as well. Overnight cultures were re-inoculated on the Luria-Bertani agar (Hi-Media, Mumbai, India), which gives excellent growth. Later, these cultures were grown overnight in the NB and checked for viability and density of the bacterial cultures by adjusting to 0.5 McFarland standard (Acharya et al., 2020). In parallel, the autoclaved NA/sterilized Mueller–Hinton agar (Hi-Media, Mumbai, India) plates were prepared for antibacterial activity by using earlier standardized protocols (Archana et al., 2021; YadavReddy et al., 2021; Ananda et al., 2022). After the solidification of the media, individual 25 µL of overnight cultures were spread on the agar plate by an L-shaped spreader. This step was repeated for both samples across four strains of bacteria used. As the methodology employs paper discs that are labeled with their respective concentration. In the current study, we used paper discs that were imbibed with 50 μL of diluted samples, and the paper disc was partially air-dried and placed equidistantly on the inoculated plates. These antibacterial plates also had paper discs soaked in DMSO, assisted as a negative control, and amoxicillin (30 mcg/disc) (Hi-Media, Mumbai, India) as a positive control. After 24–36 hrs, antibacterial activity was evaluated by measuring the diameter of the clear zone of inhibition around the discs. The assay was repeated thrice. Antibacterial activity was expressed as the mean zone of inhibition diameters (mm) produced by three NPS.

## 3 Result and discussion

### 3.1 Powdered X-ray diffraction (PXRD)

**FIGURE 2:**
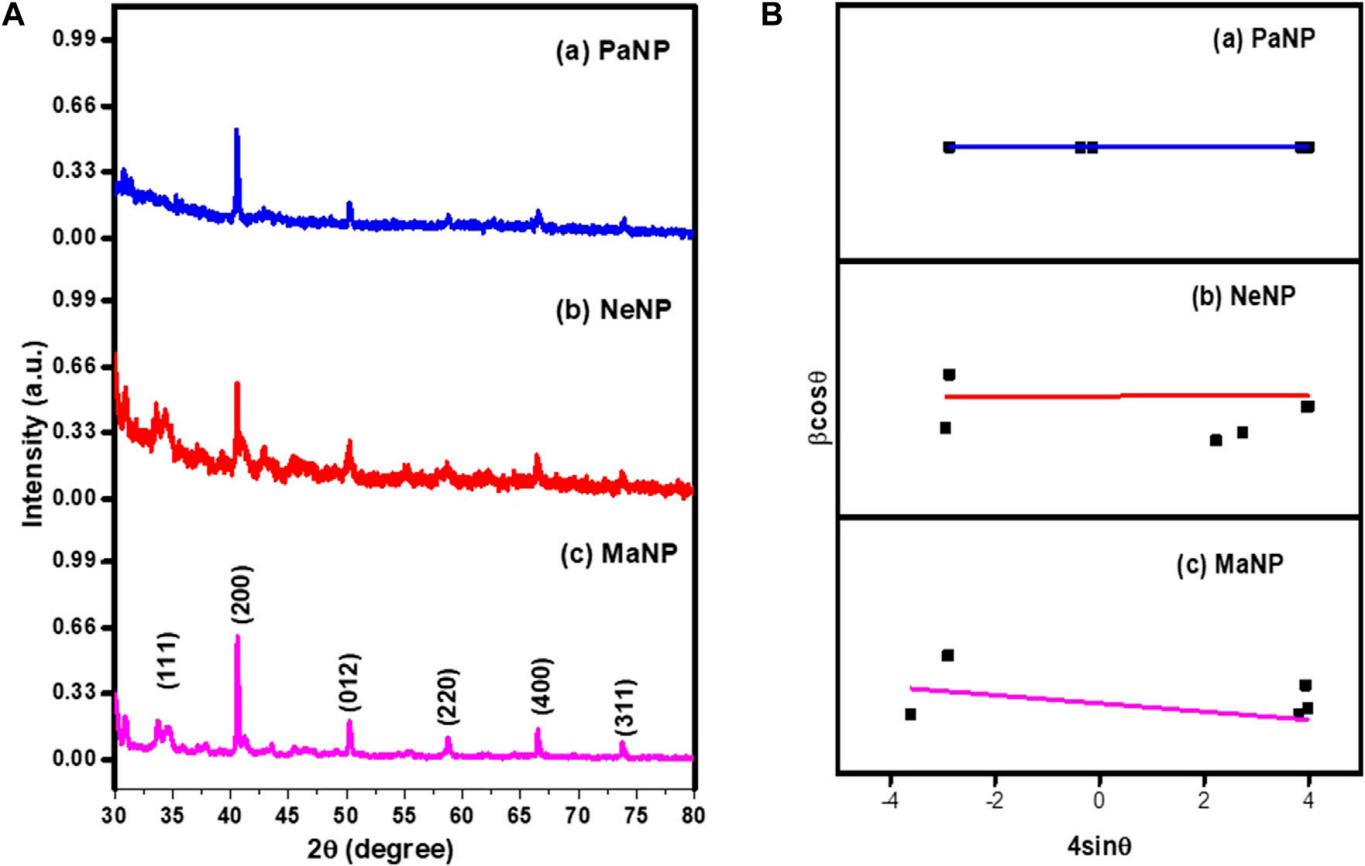
**(A)** PXRD pattern and **(B)** W-H plot of Pa, Ne, and Ma nanostructures.

The PXRD analysis of synthesized MgO nanoparticles was performed to study the crystalline structure, size, and phase purity of the sample. From Figure 2. The PXRD pattern was recorded for MgO nanostructures prepared using different plant extracts such as Ma, Ne, and Pa. The prepared MaNP, NeNP, and PaNP nanostructures exhibited peaks at (1 1 1), (2 0 0) (2 2 0), (4 0 0), and (3 1 1) planes respectively. The diffraction peaks were in accordance with the JCPDS No. 87–0653 exhibiting polycrystalline cubic structure (Diachenko et al., 2016). The reducing elements such as oxygen, hydrogen, and carbon elements in Ma and Ne might not be sufficient to complete the reaction. Hence, PaNP samples have fewer impurities than MgO prepared by Ma and Ne. In addition, these impurity peaks might be due to the phytochemicals present in the leaf extract. These extracts have a major impact on the size, structure, and phase of MgO nanoparticles. The average crystallite size and strain of the particles were estimated by Williamson–Hall (W-H) plot method (Williamson and H Hall, 1953). The crystallite size and strain values were estimated for MgO nanoparticles prepared using Ma, Ne, and Pa extracts are given in Table 1. From Table 1 it is observed that the crystallite size varies for MgO prepared by using different plant extracts that induce strain during the material preparation. The biosynthesis yields nanoparticles with different sizes and structures–Pa-derived NPs is 20.82 nm; Ne-derived NPs is 27.08 nm and Ma-derived NPs are 10.25 nm in size.

**TABLE 1:** Crystallite size and strain values estimated for MgO nanoparticles.

MgO	Crystallite size (nm)	Strain (10^−3^)
PaNP	20.82	2.778
NeNP	27.08	1.867
MaNP	10.25	3.741

### 3.2 Fourier infrared reflectance spectroscopy (FTIR)

**FIGURE 3:**
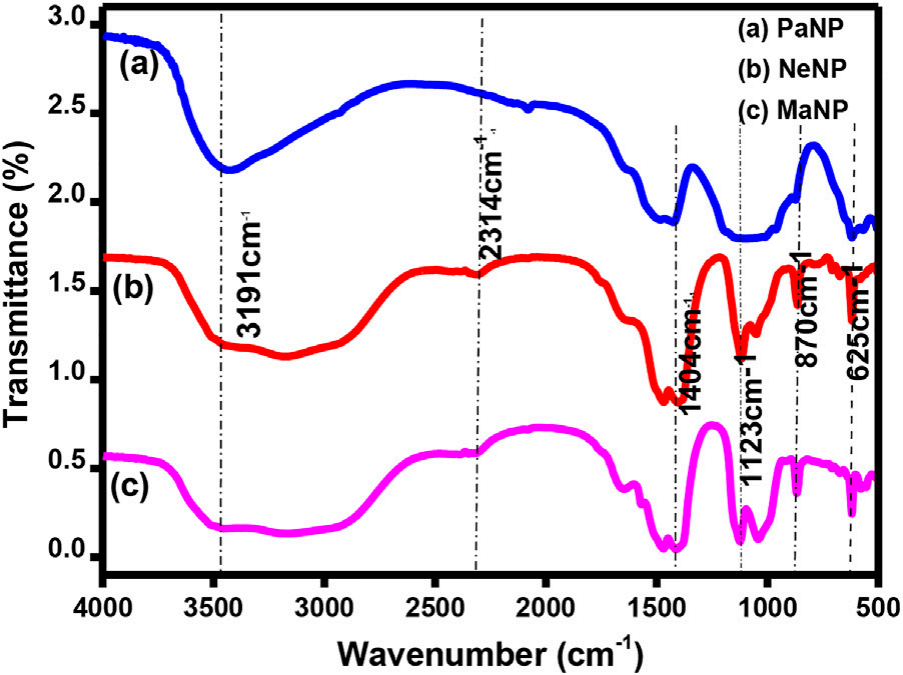
FTIR Spectra of Pa, Ne, and Ma nanostructures.

FTIR analysis determines the functional groups such as phenolic groups and metal ion bonding. Figure 3 shows the FTIR spectra of MgO nanostructures prepared using Pa, Ne, and Ma. The absorption peak observed at 3,182, 3,191 and 3,427 cm^−1^ respectively correspond to hydroxyl stretching O-H bond. The peak at 2,318 and 2,314 cm^−1^ in MaNP and NeNP respectively corresponds to C-H stretching bond of aromatic aldehyde. Peak absorbed at 1,615 cm^−1^ in MaNP corresponds to C = O carbonyl groups and 1,048 cm^−1^ corresponds to C-O indicating the presence of saturated primary alcohol (P. Y Wu, 2016). The peak absorbed in the range 1,404–1,422 cm^−1^ corresponds to αCH_2_ bending, indicating aromatic tertiary amine group/aldehyde and ketones. The Absorption peak at 1,137 and 1,123 cm^−1^ in MaNP and NeNP respectively indicate the presence of carboxylic acid, ester and alcohol. The absorption peak in the range 625–611 cm^−1^ and 870–861 cm^−1^, corresponds to Mg-O indicating the presence of MgO nanoparticles (Durgalakshmi et al., 2019; Wong et al., 2020). The comparative table indicating the presence of metals, carboxyl, and alcohol is given in Table 2.

**TABLE 2:** The absorption peaks recorded for Pa, Ne and Ma nanostructures.

Chemical bonding	PaNP	NeNP	MaNP
Mg-O	611 cm^−1^	620 cm^−1^	625 cm^−1^
Mg-O	870 cm^−1^	870 cm^-1^	861 cm^−1^
C-O (Stretching)	—	1123 cm^−1^	1137 cm^−1^
ΑCH (Bending)	1422 cm^−1^	—	1413 cm^−1^
C-H (Bending)	1480 cm^−1^	1480 cm^-1^	1476 cm^−1^
C-O (stretching)	—	—	1048 cm^−1^
C=O (stretching)	—	—	1615 cm^−1^
C-H (stretching)	—	2314 cm^−1^	2318 cm^−1^
C=C (stretching)	2073 cm^−1^	—	—
O-H (Stretching)	3427 cm^−1^	3191 cm^−1^	3182 cm^−1^

### 3.3 Scanning electron microscope (SEM)

**FIGURE 4:**
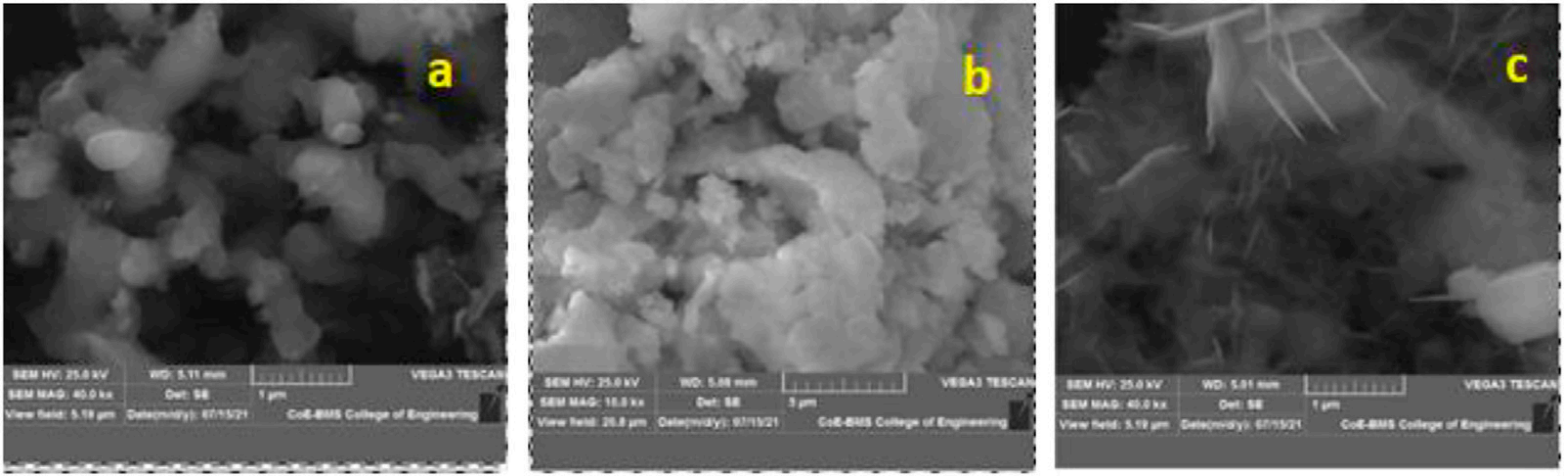
SEM images of MgO nanostructures prepared using **(A)** Pa, **(B)** Ne, and **(C)** Ma plant extracts.

The surface morphology of MaNP, NeNP, and PaNP nanostructures are shown in Figures 4A–C. The MgO nanoparticles exhibit varied morphological features due to their varied carboxyl and hydroxyl compound composition in plant extracts. Pa extract-derived samples exhibit spherical structured particles, whereas Ne extract-derived samples showed fused irregularly shaped particles, and a thin irregular flake-like structure was observed in Ma derived MgO samples.

### 3.4 UV- visible spectroscopy

**FIGURE 5:**
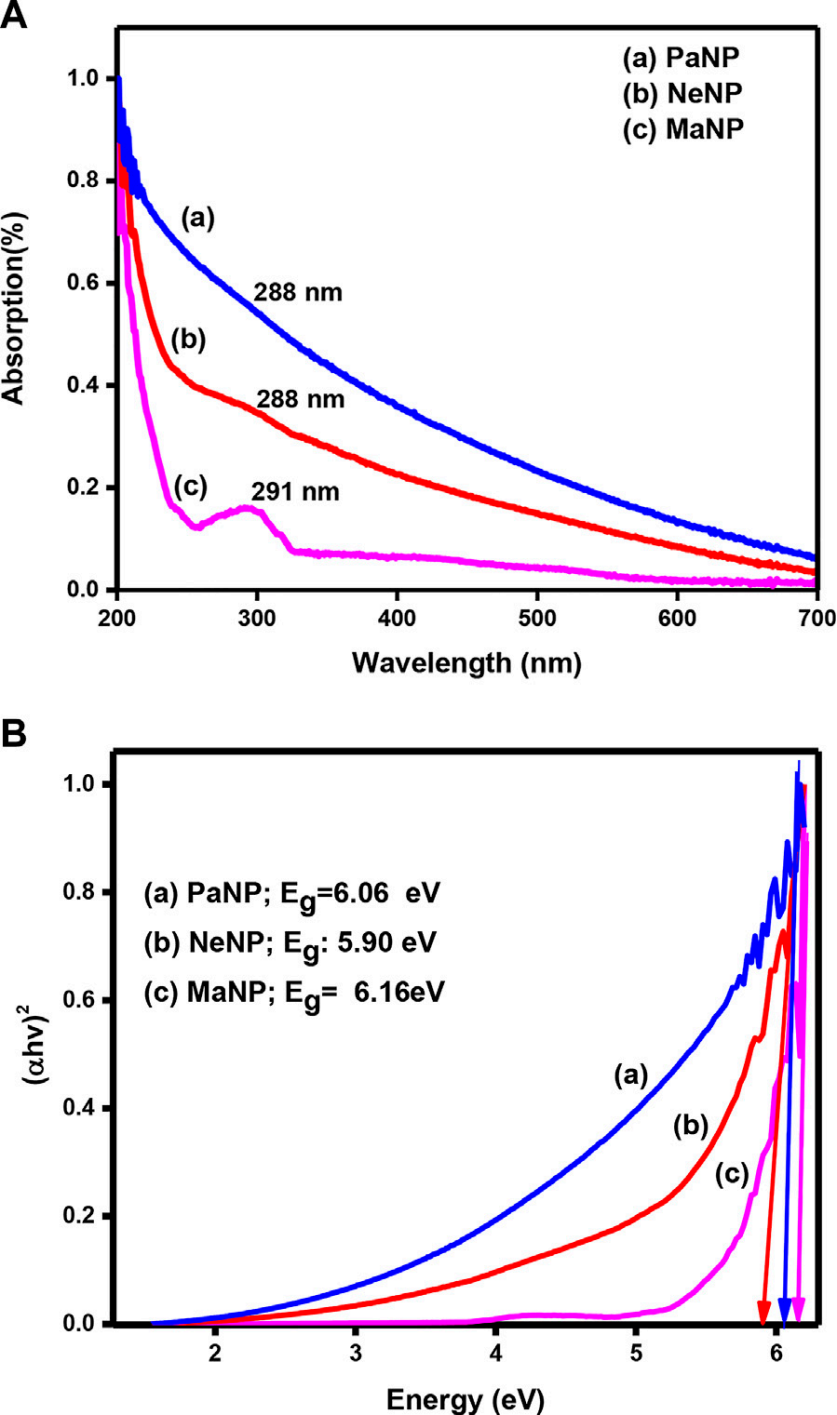
**(A)** UV-Visible absorption spectra and **(B)** Tauc plot of MgO nanoparticles prepared using Pa, Ne*,* and Ma plant extract.

The optical property of a metal nanoparticle strongly depends on the size, shape, and interaction between the particles present on the surface of the material. Absorption spectra of Mgo nanostructures were recorded at room temperature in the range 200–800 nm and the same is shown in Figure 5A. The absorption peak for MgO prepared by leaf extract of Ma, Ne, and Pa was observed at 291, 288, and 288 nm respectively. Further, the energy band gap was determined by Tauc and Wood plot equation (Nagaraja et al., 2020).

$${\left(\alpha h\nu \right)}^{1/r}=\beta \left(h\nu -E_{g}\right)$$ (1)

where, α-absorption coefficient; β-constant, h- Planck’s constant, ν-absorption frequency E.g., is the energy band gap r = 1/2 for direct band gap, r = 2 for indirect band gap.

Figure 5B shows the Tauc plot of MgO nanostructure prepared by using different plant extracts. The energy band gap determined for MgO nanostructures prepared by using Pa, Ne*,* and Ma is 6.06, 5.90, and 6.16 eV respectively. The variation in the energy band gap might be due to the change in size and shape of the particle.

### 3.5 Thermoluminescence (TL) studies

A detailed study of TL properties in MaNP, NeNP, and PaNP nanostructures was made for gamma ray irradiated samples in the dose range of 10–50 KGy. Then, the irradiated samples were used for recording the TL glow curves in the temperature range of 40–450°C at a heating rate of 3 Cs^−1^

**FIGURE 6:**
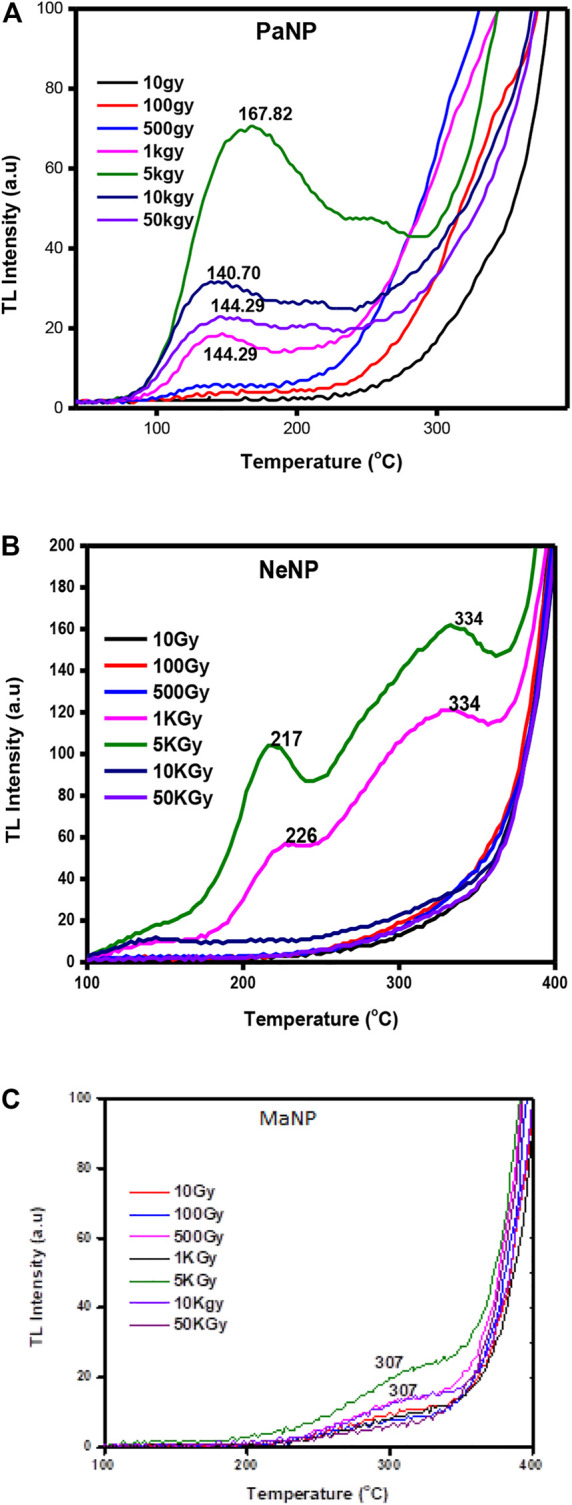
Thermoluminescence glow curve of **(A)** PaNP, **(B)** NaNP, and **(C)** MaNP at dose range from 10 to 50 KGy.

Figures 6A–C. Shows the TL glow curves of MgO nanoparticles–MaNP, PaNP, and NeNP, irradiated in the dose range 10–50 KGy. The MaNP did not exhibit any prominent glow peak other than a hump-like structure in the temperature range of 290–300°C. This indicates the formation of a shallow traps in the material. Whereas the NeNP samples showed two glow peaks at 217 and 334°C observed in 1 and 5 KGy, whereas a single glow peak at 143°C for lower and higher doses. The PaNP shows a prominent TL peak at 167°C and a shoulder peak at 217°C.

From Figure 6, it is clear that the nature of the glow curve depends on the type of fuel used during material preparation. The difference in the glow curve structure might be due to the creation of different trap location centers and defects during the material formation (Abdoul et al., 2020). Mango leaves extract tends to create very less defects, and there were no prominent glow peaks where observed.

**FIGURE 7:**
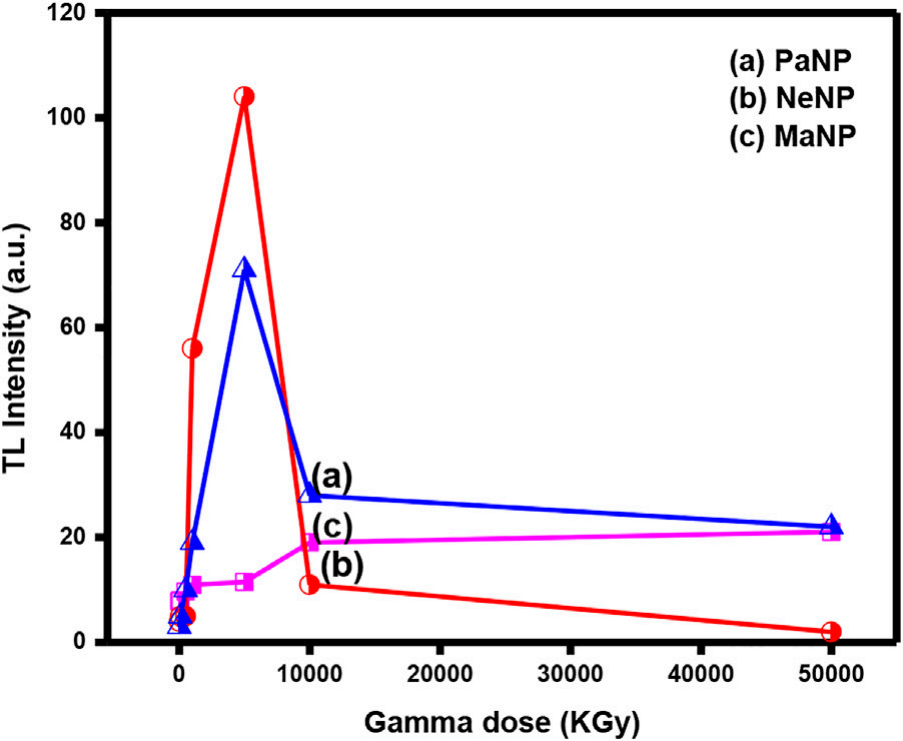
Variation of TL intensity with gamma dose in PaNP, NeNP, and MaNP.

Figure 7. Represents the variation of TL glow curve intensity variation with Co-60 gamma dose in PaNP, NeNP, and MaNP samples. It is observed that the TL glow curve intensity varies linearly up to 5 KGy and decreases with further increase in the gamma dose. As the gamma dose increases the trap centers get filled with charge carriers, further due to thermal simulation the trapped charge carriers become free and recombine with their counterparts at the recombination centers. However, at a certain optimum dose value, all the charge carriers get released and there are no more charge carriers for ionization (Souadi et al., 2022).

### 3.6 Heating rate effect

**FIGURE 8:**
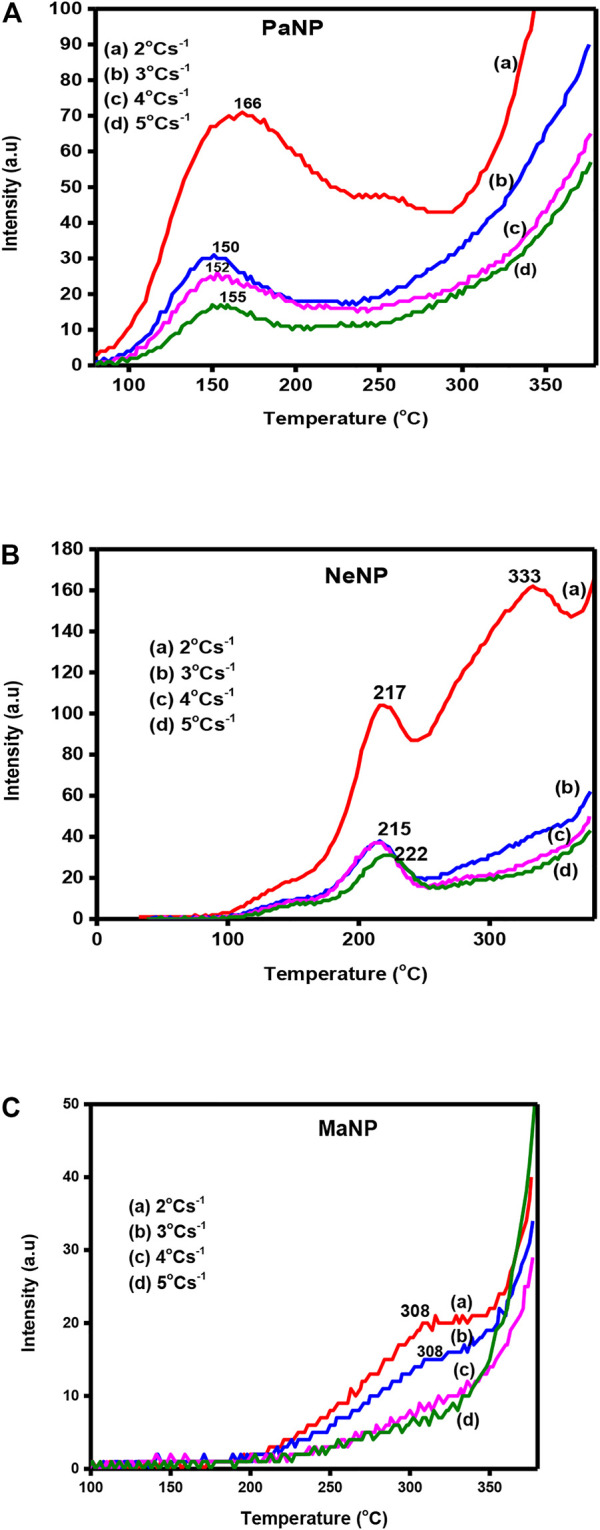
The TL glow curves recorded for different heating rate in **(A)** PaNP, **(B)** NeNP, and **(C)** MaNP.

Figure 8. Shows the TL glow curves of PaNP, NeNp and MaNP recorded for 5 KGy gamma ray irradiated samples at different heating rates varying from 2–5 Cs^−1^ range. It was observed that the glow peak temperature slightly shifted towards higher temperatures, and the area under the curve decreases. In PaNP, the structure of the glow curve slightly varies and higher temperature glow peak vanishes as the heating rate increasesas shown in Figure 8C. A similar kind of behavior was recorded in 5.12 Gy irradiated MgO: Al^3+^, Li^+^ where the glow curves are shifted to higher temperatures and the intensity is quenched with increments to the heating rate (Mofokeng et al., 2020). The shifting of the glow curves was attributed to temperature lagging, and the lowering of intensity to thermal quenching (Dogan et al., 2017; Singha and Kaintha, 2018).

### 3.7 Thermal cleaning

**FIGURE 9:**
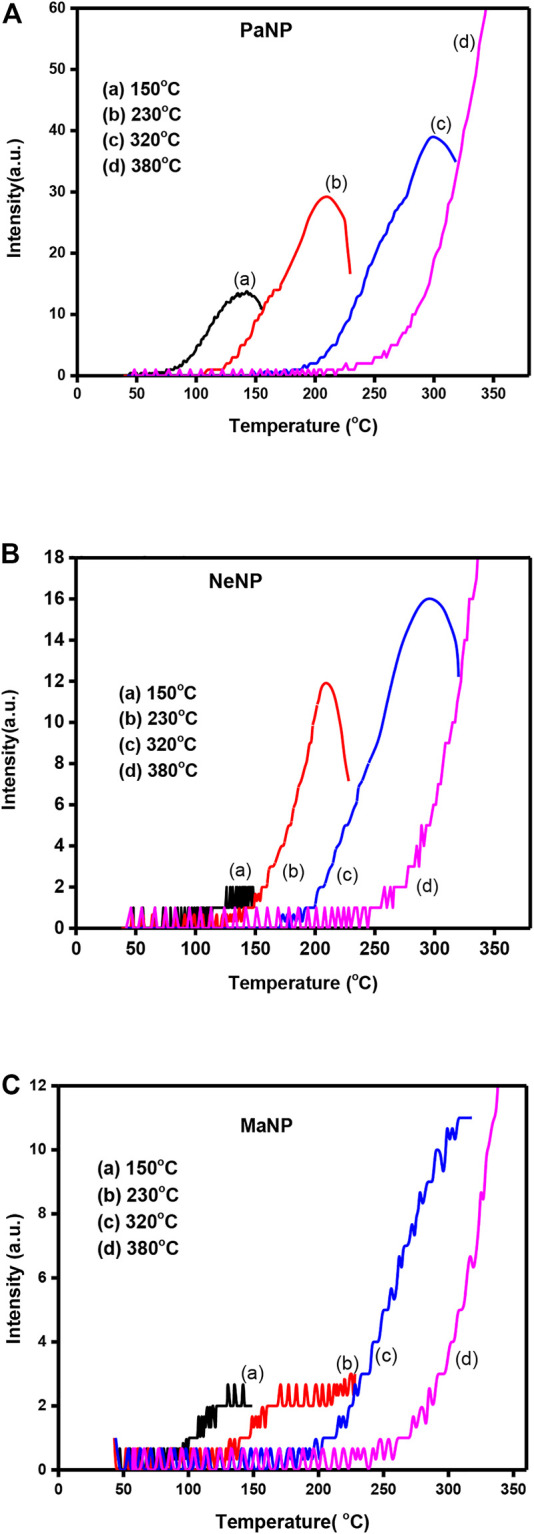
Thermally cleaned TL glow curves of **(A)** PaNP, **(B)** NeNp, and **(C)** MaNP.

The thermal cleaning method is used to resolve the overlapped peaks in the glow curves. The isolated glow peaks of 5 KGy gamma ray irradiated PaNP, NeNP and MaNP are shown in Figure 9. The phosphor material showing a simple glow curve structure has two or more peaks overlapped. Each peak has a maximum temperature at T_1_ < T_2_< … <T_n_ (Manjunatha et al., 2013). The sample is heated at a temperature beyond the maximum temperature of the first peak and cooled immediately. This method removes all the traps which lead to the first peak. This method was repeated for consisted peaks.

### 3.8 Kinetic parameter of TL glow curve

**FIGURE 10:**
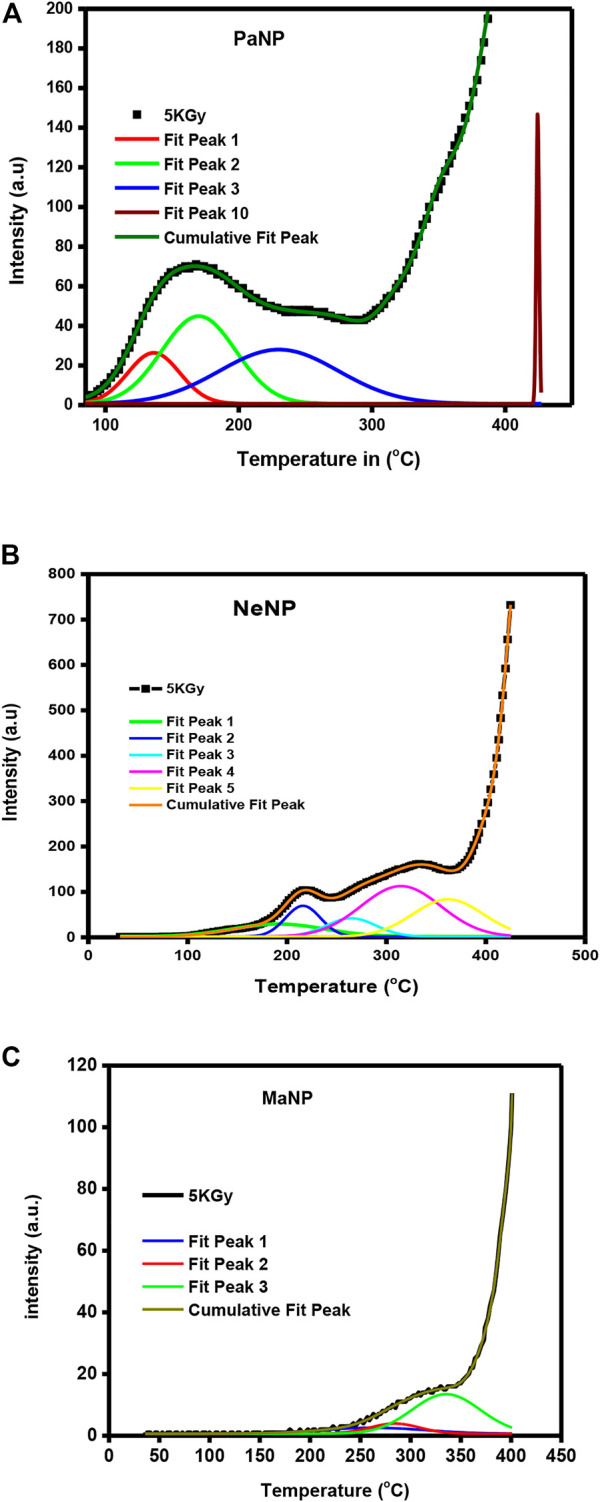
Deconvoluted glow peaks of **(A)** PaNP; **(B)** NeNP and **(C)** MaNP.

The deconvolution of glow curves was carried out using ORIGIN 9 software in support of the thermal cleaning method to disclose that it contains multiple TL trap depths (Jacob and Isac, 2014; Ramakrishna et al., 2014). And the deconvoluted glow curves of 5 KGy irradiated PaNP, NeNP and MaNP are shown in Figure 10. Further, the deconvoluted peaks were utilized for the estimation of trapping parameters using Chen’s peak method (Premkumar et al., 2012).

The activation energy(E) and frequency factor (s) (Furetta, 2003; Ramakrishna et al., 2014) were determined using the equations given below

$$E=C_{\gamma }\left(\frac{kT_{m}^{2}}{\gamma }\right)-b_{\gamma }\left(2KT_{m}\right)$$ (2)

$$\frac{\beta E}{k{T_{m}}^{2}}=s\exp\left\{\frac{-E}{k{T_{m}}^{2}}\right\}\left[1+\left(b-1\right){∆}_{m}\right]$$ (3)

The order of kinetics (b) was determined by calculating the geometrical shape factor (μ_g_)

$$\mu_{g}=\frac{\left(T_{2}-T_{m}\right)}{\left(T_{2}-T_{1}\right)}$$ (4)

where γ represents τ, δ and ω

$$\begin{array}{c} \tau =T_{m}-T_{1},\delta =T_{2}-T_{1},\omega =T_{2}\;–T_{1} \end{array}$$ (5)

b and C_γ_ are constants that depend on the order of kinetics and glow curve temperature width.

$$\begin{array}{c} C_{\tau }=1.510+3.0\left(\mu_{g}-0.42\right);b_{\tau }=1.58+4.2\left(\mu_{g}-0.42\right) \end{array}$$ (6)

$$\begin{array}{c} C_{\delta }=0.976+7.3\left(\mu_{g}-0.42\right);b_{\delta }=0 \end{array}$$ (7)

$$\begin{array}{c} C_{\omega }=2.52+10.2\left(\mu_{g}-0.42\right);b_{\omega }=1 \end{array}$$ (8)

T_1_ and T_2_ are the temperature values of half intensity at the low and lower half intensity of the glow curve. T_m_ is the temperature of the maximum intensity, β and K they are heating, rate, and Boltzman constant values. The kinetic parameters estimated are given in Table 3.

**TABLE 3:** Estimated kinetic parameters of Pa, Ne, and Ma nanostructures.

MgO	Peaks	Tm	μg	B	Eave (eV)	S (102) (s-1)
PaNP	1	137	0.48	2	0.746	59.09
	2	174	0.52	2	0.665	32.22
	3	232	0.47	2	0.688	24.84
NeNP	1	189	0.48	2	4.027	5.93
	2	216	0.5	2	5.837	11.13
	3	265	0.5	2	7.404	12.05
	4	314	0.5	2	9.078	12.83
	5	363	0.48	2	10.694	13.1
MaNP	1	336	0.48	2	0.642	11.49
	2	285	0.48	2	0.599	12.16
	3	262	0.5	2	0.234	8.3

### 3.9 Fading

**FIGURE 11:**
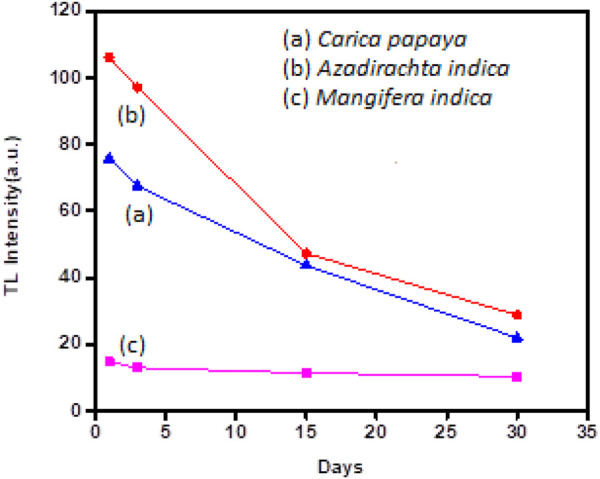
Fading characteristics shown in PaNP, NeNP, and MaNP.

Fading is one of the major properties in studies of dosimetry applications, which shows the loss of TL signal with time. The charge carrier may escape from their trap centres at room temperature due to thermal fading. It is said to be an incredibly sensitive material that must be stored with extreme caution in a container that is entirely closed to avoid fading from outside light sources. The transition between localised bands at recombination sites, which results in the tunneling of the trapped charges, is another source of fading. Figure 11 shows variation of glow peak intensity with days in MaNP, NeNP, and PaNP samples irradiated at 5 KGy gamma irradiation. It is observed that MaNP shows high fading of 70% where as NeNP and PaNP shows less fading of 27% and 30% respectively.

Thermoluminescence properties of different rare Earth or transition metal ion-doped nanomaterials have been studied by several researchers. The materials such as MgO: Ca^2+,^ Eu, Dy, and Sm doped La_2_MoO_4_, SrGd_2_O_4_:Sm^3+^, Ca_2_Al_2_SiO_7_: Dy^3+^, Al_2_O_3_: C and Al_2_O_3_:Cr, Ni, ZnO; Gd^3+^ have shown their capability of capturing electrons/holes in the forbidden band due to the presence of dopant ion, particularly suitable to the development of materials for thermo-luminescent dosimeters (Kumamoto et al., 2018; Isik and Gasanly, 2019; Ayvacikli et al., 2020; Sarasola-Martin and CorrecherGarcia-Guine, 2021; Saraswathi et al., 2022; SarıkcıTopaksuBakrCan, 2022). Our results are in agreement with these works, confirming that the nanoparticles synthesized using PaNP plant extracts are mostly suitable for Dosimetric applications.

### 3.10 Antibacterial activity

**FIGURE 12:**
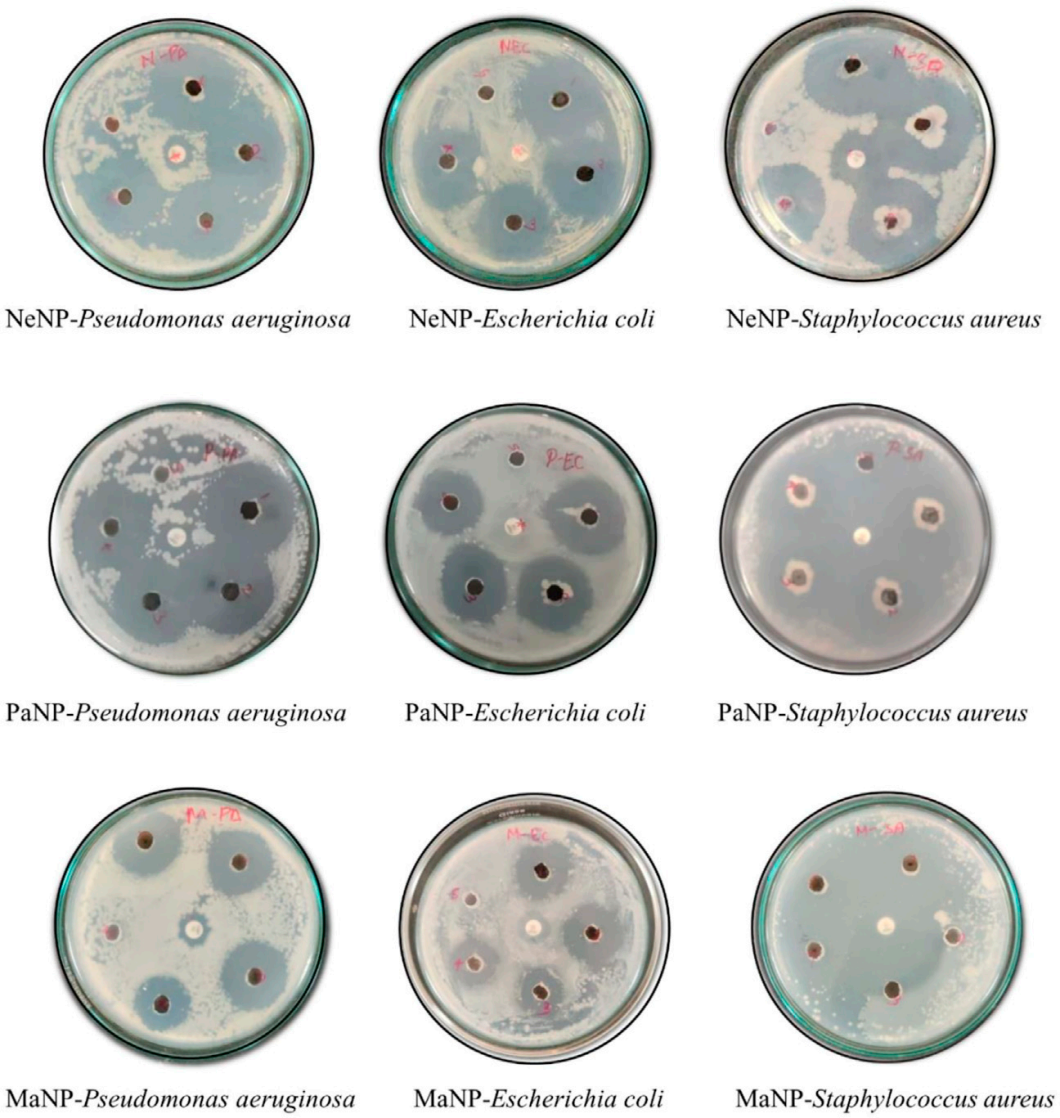
Zone of inhibition of three microorganisms by the naturally synthesized NPs from neem, papaya, and mango which are named NeNP, PaNP, and MaNP, respectively. Dilution used 1–200 mg/mL; 2–100 mg/mL; 3–50 mg/mL; 4–25 mg/mL; 5-negative control (autoclaved distilled water) and center amoxicillin -positive control.

**FIGURE 13:**
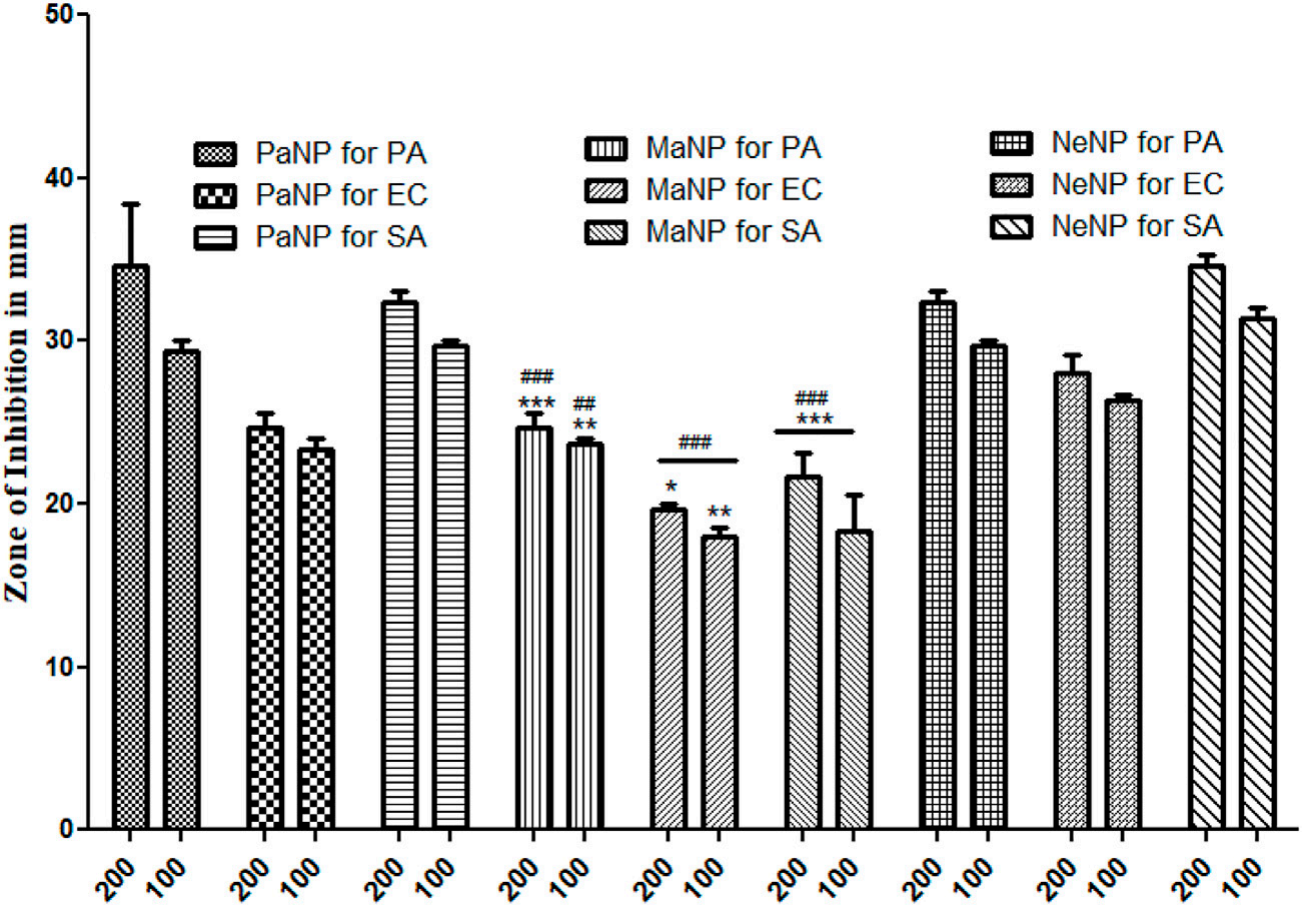
Antimicrobial activity of naturally synthesized NPs from neem, papaya, and mango which are named NeNP, PaNP, and MaNP, respectively against 3 pathogenic strains by Gel diffusion method. *Pseudomonas aeruginosa* (PA) *Escherichia coli* (EC) and Gram-positive *Staphylococcus aureus* (SA) (Values represent the Mean S.E. for n = 4 replicates. [***] *p* < 0.001; [**] *p* < 0.01 and [*] *p* < 0.05 compared PaNP with two concentration (200 and 100) all other pathogenic strains used in the study. [^###^] *p* < 0.001 and [^##^] *p* < 0.01 compared NeNP with two concentration (200 and 100) all other pathogenic strains used in the study.

The results were interpreted depending on the clearance zone seen in each of the particulates. The current analysis in Figure 12, demonstrated excellent inhibition by PaNP and NeNP in comparison to MaNP across all pathogenic microbes used in the study (Adarsha et al., 2022). However, a higher concentration of both the samples showed a zone of clearance for Gram-Positive and Gram-Negative with *Escherichia coli* lower compared to the o other two. The study also highlighted the remarkable inhibition of *Staphylococcus aureus* with the use of natural NPs, as an effective bactericidal agent. Figure 13 depicts the comparison of the zone of inhibition by the synthesized MgO NPs. Area of inhibition seen the media indicates the death or lysis of bacterial cell due to action of NPs used in the current study. This is the place where bacteria are unable to grow or become static with the given NP at minimum inhibitory concentration which prevents visible growth of a microorganism after overnight incubation with media similar to several of antibiotics used in the treating infections (Jadimurthy et al., 2022).

The effect of the nanoparticles on each of the selected pathogenic bacteria is compared and it was found that PaNPs are indicating lower rate of inhibition against *Pseudomonas aeruginosa* compared to NeNP and MaNP at all the four different concentrations—25, 50, 100 and 200 mg/mL. PaNP and NeNP are showing similar inhibition rate against *E. coli*. MaNP is comparatively showing less inhibition rate. The inhibition rate against *S. aureus* is higher in NeNP followed by PaNO and MaNP. This study implies that the Pa-derived and Ne-derived MgNPs are effective antibacterial potency against the human pathogens, as compared to MaNPs.

## 4 Conclusion

Ma*,* Ne*,* and Pa were used as reducing agents during the preparation of MgO nanostructured material by using a simple solution combustion method. The 500°C calcinated MgO nanostructure exhibited a well-defined diffraction pattern having a cubic structure and crystallite size varying between 10.25–27.08 nm. FTIR spectra revealed the stretching bond corresponding to Mg-O, carboxyl, and hydroxyl groups. The effect of reducing agents on morphological features was observed in SEM micrographs. MaNP, NeNP, and PaNP exhibited irregular flakes, fused particles, and spherical structured particles respectively. Further, the energy band gap estimated using by Tauc plot was found to be 6.16, 5.90, and 6.06 eV for MaNP, NeNP, and PaNP respectively. Well-defined TL glow curves were observed in PaNP at 167°C to 217°C and NeNP at 217°C and 334°C respectively. The good linear behavior, less fading, and simple glow curve structure are the important characteristics to be used for dosimetric applications. Amongst, PaNP nanostructure exhibits good TL behavior when compared to NeNP and MaNP. Hence, PaNP can be used as a potential material for dosimetry application. In this study, we also observed a better bacteriostatic potency of both PaNP and NeNP at 200 and 100 μg/mL by blocking the growth of communalistic pathogen nanoparticles. This trend of growth inhibition seen by a zone of clearance can be strongly correlated to the death of the colonies mediated by NPs. Many studies have revealed that NP-mediated bacterial inhibition may be due to leaky walls, damaged genetic material, malfunctioning of cell organelles, lipid peroxidation, and many more owed by free radicals triggered by NPs. Our investigation reveals the use of green synthesized as the replacement antibiotics (Saran et al., 2021), (Bos et al., 2006), (Krishna Reddya et al., 2018).

## Data Availability

The raw data supporting the conclusion of this article will be made available by the authors, without undue reservation.
